# A rare case of poorly differentiated thyroid carcinoma probably arising from a nodular goiter

**DOI:** 10.1186/s12907-017-0048-x

**Published:** 2017-06-02

**Authors:** Hironao Yasuoka, Yasushi Nakamura, Mitsuyoshi Hirokawa, Ken-ichi Yoshida, Kana Anno, Masayuki Tori, Masahiko Tsujimoto

**Affiliations:** 1 Department of Pathology, Osaka Police Hospital, 10-31 Kitayama-cho, Tennouji-ku, Osaka City, Osaka, 543-0035 Japan; 2 Department of Pathology, Osaka Cytopathological Laboratory, 2-2-26, Kunijima, Higashiyodogawa-ku, Osaka City, Osaka, 533-0024 Japan; 3 Department of Pathology, Kuma Hospital, 8-2-35, Shimoyamate-dori, Chuo-ku, Kobe City, Hyogo 650-0011 Japan; 4 Department of Pathology and Clinical Laboratories, National Cancer Center Hospital, 5-1-1, Tsukiji, Chuo-ku, Tokyo, 104-0045 Japan; 5 Department of Surgery, Osaka Police Hospital, 10-31 Kitayama-cho, Tennouji-ku, Osaka City, Osaka, 543-0035 Japan

**Keywords:** Poorly differentiated thyroid carcinoma, Nodular goiter, *RAS* mutation

## Abstract

**Background:**

Some poorly differentiated thyroid carcinomas (PDTC) arise from pre-existing, well-differentiated carcinomas of follicular cell origin; however, others most likely arise de novo*.* The case of a PDTC adjacent to a pre-existing nodular goiter is very rare.

**Case presentation:**

A patient had a PDTC, a widely invasive, cellular tumor with cells that lacked the nuclear features of a papillary thyroid carcinoma. Carcinoma cells were arranged in trabecular, solid, and microfollicular histological patterns and displayed high mitotic activity. A nodule partially encapsulated in a thick fibrous capsule was found adjacent to the PDTC. The nodule was composed of small or dilated follicles, without papillary carcinoma-like nuclear features, that were consistent with a nodular goiter. The PDTC showed a high Ki-67 labeling index and an *NRAS* gene mutation (codon 61, Q61K).

**Conclusion:**

These results support our diagnosis of a PDTC, probably arising from a nodular goiter.

## Background

Poorly differentiated thyroid carcinoma (PDTC) is a rare neoplasm with an aggressiveness midway between that of differentiated (follicular and papillary carcinomas) and undifferentiated carcinomas [1]. Some PDTCs arise from pre-existing, well-differentiated carcinomas of follicular cell origin; however, others most likely arise de novo [2]. PDTC is more common in women and in patients older than 50, but may also be present in adolescents and children [3–5]. Genetic alterations in PDTC have been previously reported such as in *RAS*, *TP53*, *BRAF*, or *CTNNB1* genes [6]. These genetic alterations are also common in many cancers, such as those of the stomach, and especially cancers that develop at a young age.

In this study, we report a rare case of a PDTC arising from a pre-existing goiter and provide information on the molecular alterations of these two components.

## Case presentation

**Fig. 1:**
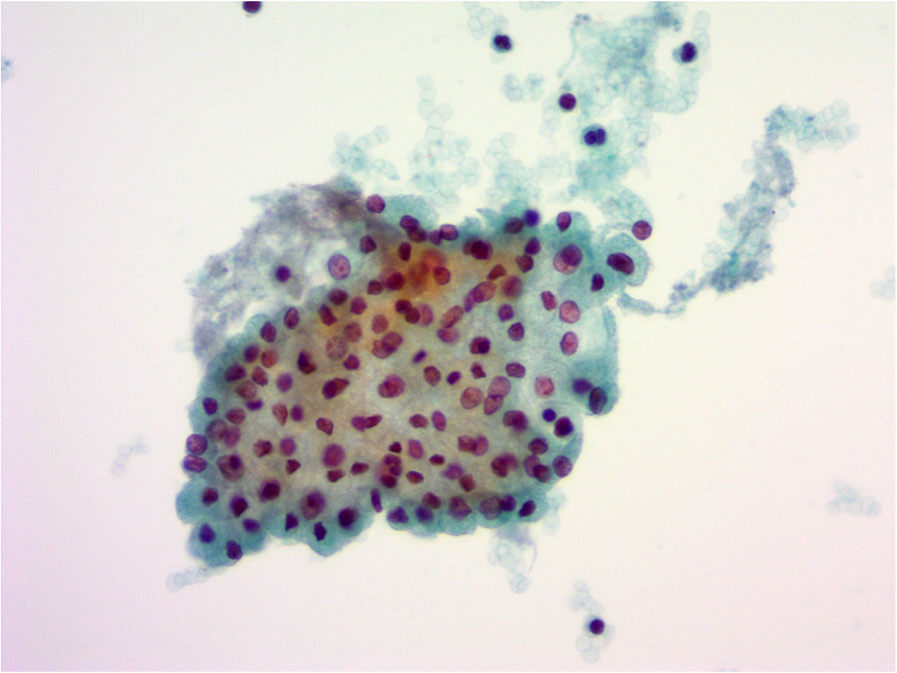
Microscopic appearance of a fine-needle aspiration biopsy and cytological examination of the thyroid. Follicular cells are arranged as sheets, compatible with the smear of a nodular goiter. (Papanicolaou, ×40)

A 35-year-old female presented with a thyroid nodule as revealed by computed tomography (CT) examination during follow-up of a gastric adenocarcinoma. The patient’s past medical history included a gastric adenocarcinoma (at 29 years of age, laparoscopic assisted distal gastrectomy) and a cerebral infarction (at 35 years of age). Clinical signs of thyroid dysfunction were not obvious in a clinical exam. A cervical lesion ultrasound and CT examination revealed a left thyroid nodule, 29 mm in diameter, consisting of a non-calcified solid mass with low density. A fine-needle aspiration biopsy and cytological examination of the mass revealed follicular cells arranged as sheets or follicles, and hemosiderin-laden macrophages, compatible with the smear of a nodular goiter (Fig. 1). Fluorodeoxyglucose – positron emission tomography scanning was not performed at the initial scan. The patient was subsequently observed. Approximately 2 years later, the left thyroid nodule had progressively increased in size to become 36 mm in diameter. Without a definitive diagnosis, the patient underwent a left hemithyroidectomy with No. I and II lymph node dissections. Her recovery was uneventful. After a further pathological examination, the patient underwent a completion thyroidectomy with central lymph node dissection and her subsequent recovery was again uneventful. The patient’s condition was followed by radioactive iodine therapy after completion thyroidectomy, and external beam radiation therapy was performed. She remains in relatively good health, 10 months after the operation.

Immunohistochemical stains were performed on formalin-fixed and paraffin-embedded tissue. Four-micrometer sections were stained with antibodies against TTF-1 (Dako, Glostrup Denmark), PAX-8 (Proteintech, Chicago, IL, USA), thyroglobulin (Dako), p53 (Leica Biosystems, Wetzlar, Germany), and Ki-67 (Dako). Immunohistochemical staining was performed with an automated staining system (BenchMark ULTRA, Ventana, Tucson, AZ, USA). The primary antibody was incubated for 30 min at a dilution of 1:30 for anti–TTF-1, 1:300 for anti–PAX-8, 1:3000 for anti-thyroglobulin, 1:50 for anti-p53, and 1:50 for anti–Ki-67, at 37 °C, respectively.

DNA extraction and mutation analyses for *RAS* and *TP53* genes were conducted at LSI Medience Corporation (Tokyo, Japan). Genomic DNA was extracted from formalin-fixed, paraffin-embedded tissue. Serial slices (5 mm thickness) were made from a block for tumor cell dissection. After deparaffinization with xylene, tissue sections were stained with hematoxylin and eosin. Target tumor lesions were macroscopically dissected to minimize contamination with normal tissue. Sequencing analysis was performed for *TP53* mutations (exons 5, 6, 7, and 8), while a polymerase chain reaction–reverse sequence specific oligonucleotide method using a MEBGEN™ RASKET Kit (Medical and Biological Laboratories Co., Ltd., Nagoya, Japan) was undertaken for *RAS* mutation analysis (*KRAS/NRAS* codons 12, 13, 59, 61, 117, and 146).

**Fig. 2:**
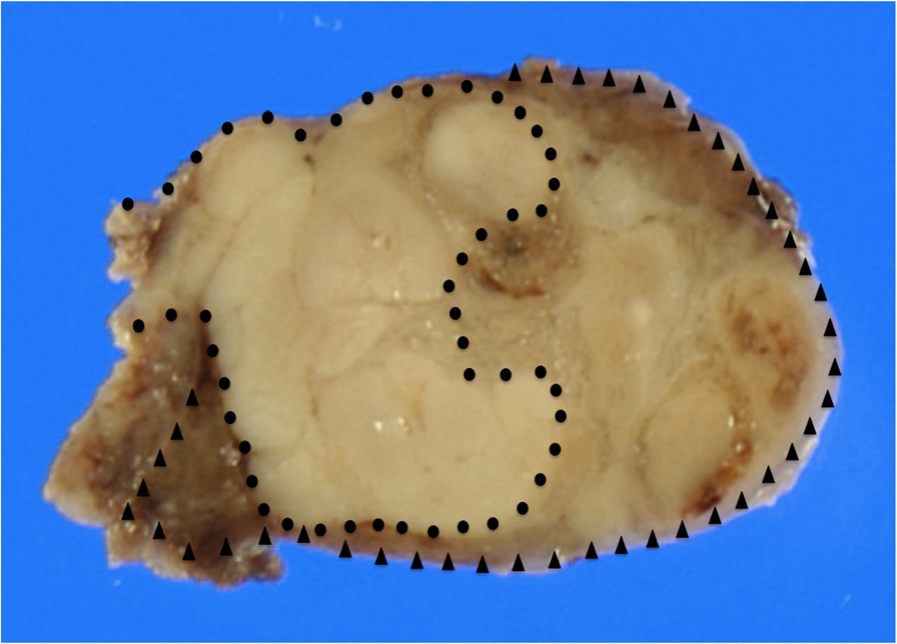
Macroscopic appearance of a nodule in thyroid tissue. The cut surface revealed a lobulated, infiltrating, and solid gray-whitish tumor, with a thick, focal capsule. Dots outline the poorly differentiated carcinoma component and the arrowheads indicate the nodular goiter

**Fig. 3:**
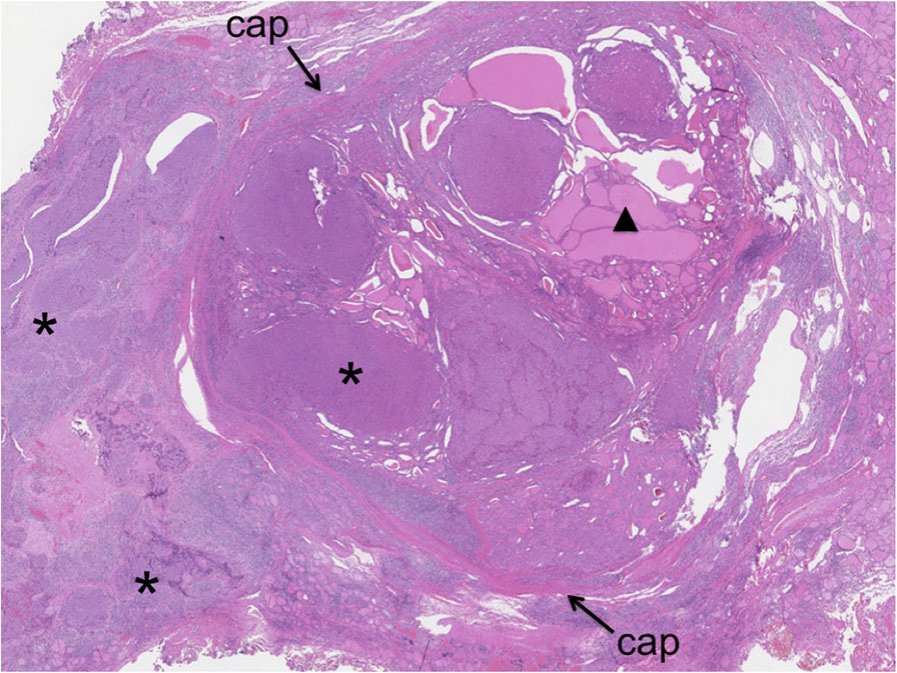
Microscopic appearance of a nodule in thyroid tissue. A widely invasive, cellular tumor (*asterisks*), with a nodular goiter (*arrowhead*) within a nodule partially encapsulated by a thick fibrous capsule (*cap*) was noted. (hematoxylin and eosin, ×1.25)

**Fig. 4:**
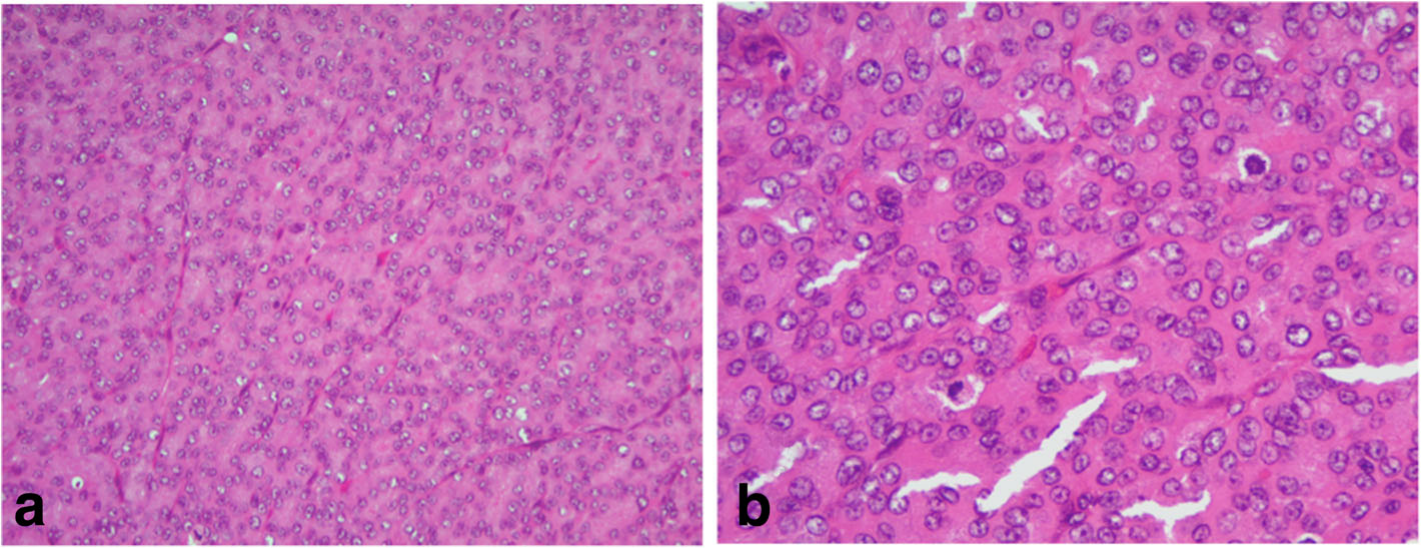
The poorly differentiated thyroid carcinoma showed trabecular, solid and microfollicular histological patterns without the nuclear features of a papillary thyroid carcinoma **a**, as well as high mitotic activity **b**. (**a**–**b**: hematoxylin and eosin, **a**: ×10, **b**: ×20)

**Fig. 5:**
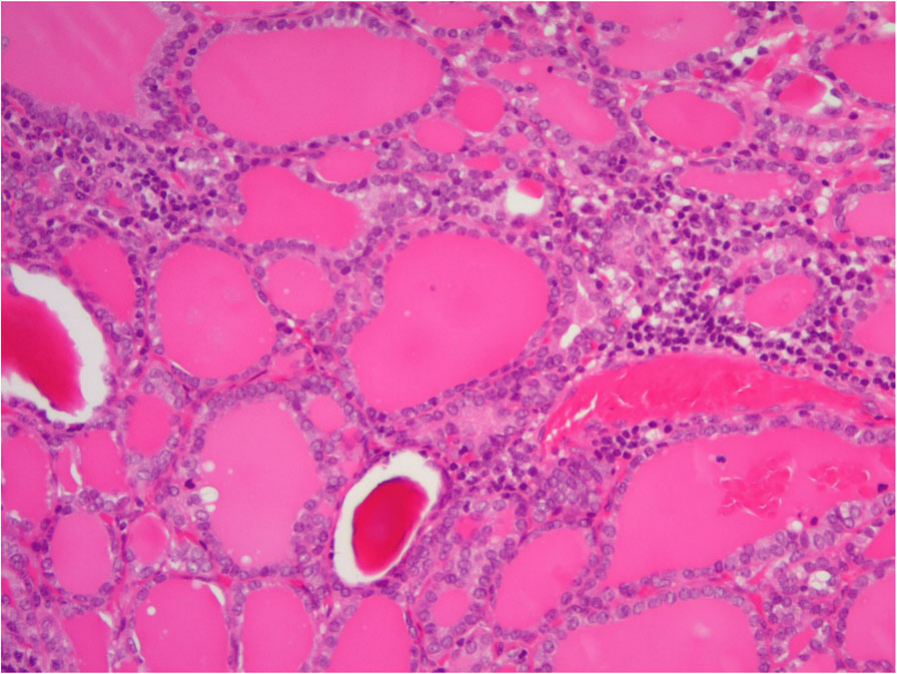
The nodular goiter was composed of small or dilated follicles, and lacked the nuclear features of a papillary thyroid carcinoma. (hematoxylin and eosin, ×10)

**Fig. 6:**
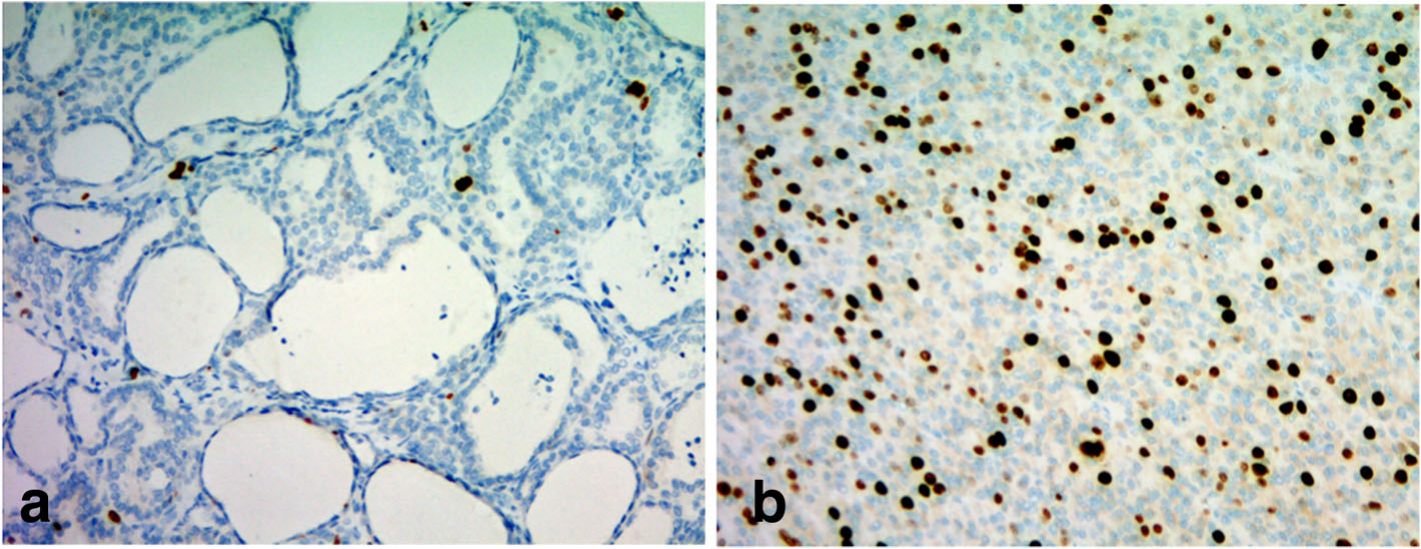
The Ki-67 labeling index of the nodular goiter was very low **a**; however, that of the PDTC was high **b**. (Ki-67 immunostain, **a**–**b**: ×10)

A 29 × 30 mm nodule was seen in the patient’s thyroid tissue. When cut, the nodule’s surface showed a lobulated, infiltrating, and solid gray-whitish tumor with a thick, focal capsule (Fig. 2). Microscopically, a widely invasive, cellular tumor (Fig. 3) was observed, with tumor cells lacking the nuclear features of a papillary thyroid carcinoma and arranged in trabecular, solid, and microfollicular histological patterns (Fig. 4a). A mitotic activity of four mitoses per 10 high-power fields was noted (Fig. 4b). Vascular invasion, tumor necrosis, and convoluted nuclei were not apparent. According to the diagnostic criteria outlined for this disease [7], a diagnosis of PDTC was made. The residual thyroid showed a nodule partially encapsulated by a thick fibrous capsule, and composed of small or dilated follicles but without papillary carcinoma-like nuclear characteristics (Fig. 5). These features were consistent with a nodular goiter, which was positive for TTF-1, PAX-8, and thyroglobulin, and negative for p53. The PDTC component of tissue was positive for TTF-1 and PAX-8, and was sparsely scattered, with immunoreactive staining for thyroglobulin and p53 present. The Ki-67 labeling index of the nodular goiter was very low (1.4%; Fig. 6a); however, that of the PDTC was high (20.5%; Fig. 6b). The proportion of PDTC area within the nodular goiter was approximately 50%. In remnant thyroid tissue of a completion thyroidectomy, a whitish 13 × 10 mm nodule was seen. Contiguous to scar formation, including a suture granuloma, residua of poorly differentiated carcinoma cells were identified. Lymph nodes were free of metastases.

DNA was extracted from the nodular goiter and PDTC, and mutation analyses for *RAS* (KRAS/NRAS codon 12, 13, 59, 61, 117, 146) and *TP53* (exon 5, 6, 7, 8) genes were performed as described above. For the *RAS* oncogene, the *NRAS* gene in codon 61, a Q61K mutation, was seen in the PDTC portion of tissue only. Other *RAS* or *TP53* gene mutations were not detected in the nodular goiter or PDTC.

## Discussion and conclusions

PDTC is a neoplasm that accounts for only 4–7% of all thyroid malignancies [1]. Because of its rare occurrence [8] and the heterogeneity of inclusion criteria [1, 3, 7, 9–11], a diagnosis of PDTC is difficult. Most PDTCs arise from a follicular or papillary thyroid carcinoma, or are de novo; rarely do they arise from a nodular goiter.

The present case describes a widely invasive, cellular tumor with cells lacking the nuclear features of a papillary thyroid carcinoma that were arranged in trabecular, solid, and microfollicular histological patterns. Since a high mitotic number and Ki-67 labeling index were also identified, a diagnosis of PDTC was made. Other results from an immunohistochemical panel were consistent with a diagnosis of PDTC. The residual thyroid showed a nodule that was incompletely encapsulated by a fibrous capsule and that varied in thickness. Microscopically, the nodule was composed of small, slightly cystic follicles that lacked papillary carcinoma-like nuclear characteristics. The histopathological features of the residual thyroid were compatible with a nodular goiter. Regarding a differential diagnosis for the nodular goiter or PDTC of the present case, a multinodular subtype of the follicular variant of a papillary carcinoma was considered. Because papillary carcinoma-like nuclear features were not seen in both the nodular goiter and PDTC, a diagnosis of a papillary carcinoma was excluded.

As for a differential diagnosis for the nodular goiter, a follicular carcinoma or adenoma was considered. A typical follicular adenoma is enclosed in a fibrous capsule [12] and a minimally invasive follicular carcinoma is usually well-encapsulated by a thicker and irregular capsule [13]. As the nodule in the present case was incompletely encapsulated by a fibrous capsule, a diagnosis of a minimally invasive follicular carcinoma or adenoma was ruled out. However, a widely invasive follicular carcinoma was considered. In the present case, a mutation at codon 61 of *NRAS* was detected in the PDTC portion of the patient’s thyroid tissue. *RAS* mutations have been reported with similar frequencies (ranging from 20 to 50%) in follicular carcinoma and adenoma; however, these are uncommon in nodular goiter [14]. These results support our diagnosis of a PDTC, probably arising from a nodular goiter. In terms of a differential diagnosis, we postulate an invasion of the adenomatous nodule by a PDTC. The PDTC component was widely surrounded by a nodular goiter as shown in Fig. 2, suggesting that the PDTC probably arose from a nodular goiter. A previous report has described a case of a PDTC arising in a goiter; however, to the authors’ knowledge, this is the first report of a case of PDTC arising from a goiter as defined by molecular analysis.

PDTCs occur in adults (the median age is 55 years), but may also be present in adolescents and children [3, 5]. The patient’s medical history included a gastric adenocarcinoma (at 29 years of age), with a histological subtype of a diffuse carcinoma (signet ring cell carcinoma). Gastric adenocarcinomas are rare in those aged <30 years and tumors are more likely to be hereditary in young people [15]. However, the patient’s family medical history did not include reports of any cancer. *RAS* gene mutations are typically identified in codons 12, 13, and 61, while for *NRAS*, codon 61 mutations are the most frequent; mutated *HRAS* and *KRAS* codons 12 and 13 are also observed in PDTC [6]. On the other hand, mutations of *KRAS* and *NRAS* are rare in gastric adenocarcinoma; *KRAS* (4.9%) and *NRAS* codons 12/13 (1.9%) are usually detected [16]. As the present case showed a mutation at codon 61 of *NRAS* in the PDTC portion, this suggests that genetic factors of PDTC are not associated with gastric adenocarcinoma.

Nodular goiter is a common thyroid disease. Most benign thyroid nodules grow slowly [17]; however, the rate of growth of thyroid nodules as assessed by ultrasound does not distinguish between benign and malignant thyroid nodules [18]. A previous report described how, despite an average increase in volume of about 70% during 5 years’ follow-up, only 1 of 74 reaspirated thyroid nodules was found to be malignant [17]. In another case, clinical findings that suggested a diagnosis of thyroid carcinoma in an euthyroid patient with a solitary nodule included a family history of medullary carcinoma or multiple endocrine neoplasia, rapid tumor growth, a nodule that was very firm or hard, fixation of the nodule’s adjacent structures, paralysis of vocal cords, regional lymphadenopathy, and distant metastases [19, 20]. In the present case, cytological examination of a fine-needle aspiration biopsy of the thyroid mass revealed follicular cells compatible with the smear of a nodular goiter; the patient was subsequently followed by observation. However, because of rapid tumor growth, surgery was recommended, regardless of the results of cytology. On the basis of the findings of the present case, and as mentioned previously [19, 20], we conclude that a patient with a thyroid tumor showing rapid growth should be offered surgery, regardless of the results of a fine-needle aspiration biopsy.

In conclusion, based on the patient’s medical history, the histopathological diagnosis, and the results of the immunohistochemical staining panel and molecular analysis, a PDTC arising from a nodular goiter is the most probable diagnosis in the present case.

## Abbreviations

CT: computed tomography
PDTC: poorly differentiated thyroid carcinoma
